# Measurement of crystalline lens tilt in high myopic eyes before cataract surgery using swept-source optical coherence tomography

**DOI:** 10.1186/s40662-020-00176-5

**Published:** 2020-03-06

**Authors:** Qiang Lu, Wenwen He, Dongjin Qian, Yi Lu, Xiangjia Zhu

**Affiliations:** 1 Department of Ophthalmology, Eye and Ear, Nose, and Throat Hospital, Fudan University, 83 Fenyang Road, Shanghai, 200031 People’s Republic of China; 2 Eye Institute, Eye and Ear, Nose, and Throat Hospital of Fudan University, Shanghai, 200031 People’s Republic of China; 3 Key Laboratory of Myopia, Ministry of Health, Shanghai, 200031 People’s Republic of China; 4 Shanghai Key Laboratory of Visual Impairment and Restoration, Shanghai, 200031 People’s Republic of China

**Keywords:** Crystalline lens tilt, Orientation, Magnitude, High myopia, Swept-source optical coherence tomography

## Abstract

**Background:**

To measure the crystalline lens tilt in eyes with various degrees of myopia before cataract surgery using swept-source optical coherence tomography (SS-OCT).

**Methods:**

We used SS-OCT (IOLMaster 700) to scan 131 emmetropic eyes (axial length < 24.5 mm), 25 mild/moderate myopic eyes (axial length 24.5–26 mm), and 123 high myopic eyes (52, 29, and 42 eyes with axial lengths of 26–28, 28–30, and > 30 mm, respectively) as part of the routine preoperative examination before cataract surgery. SS-OCT involved B-scans along six meridians. The data were analyzed to assess the magnitude and orientation of the lens tilt and their correlation with other optical biometric parameters.

**Result:**

The mean tilt was 3.36 ± 0.98° in emmetropic eyes, 3.07 ± 1.04° in mild/medium myopic eyes, and 2.35 ± 1.01° in high myopic eyes. Tilt correlated significantly and inversely with axial length (Pearson’s *r* = − 0.427, *P* < 0.001). The crystalline lens tilt predominantly faced the upper outer quadrant relative to the visual axis, symmetrically in both eyes, with mean angles of 24.32° and 147.36° in the right and left eyes, respectively. The variability in the lens tilt direction increased with increasing axial length (χ^2^ test, *P* < 0.001).

**Conclusion:**

The magnitude of crystalline lens tilt decreased with increasing axial length. The direction of tilt was predominantly towards the upper outer quadrant in both eyes. The variability in the tilt orientation increased with increasing axial length.

**Trial registration:**

NIH (clinicaltrial.gov), NCT03062085. Registered 23 February 2017.

## Background

The prevalence of clinically relevant myopia (more than − 1.0 diopter [D]) is increasing in recent birth cohorts in both developed and developing countries [1–7]. Data suggest that high myopia (more than − 6.0 D or an axial length [AL] of greater than 26 mm) [8, 9] is even more common in Asian populations [10].

Important differences in developmental processes and the intraocular environment have been demonstrated in high myopic and less myopic eyes. Structural alterations, including the thinning and degeneration of the eye layers [11], the overstretching of the eyeball [12], and the proinflammatory state of the aqueous humor [13, 14], are all related to abnormalities of the suspensory ligament in high myopic eyes.

Since greater decentration of the intraocular lens (IOL) has been observed in myopic eyes after surgery [15] and the postoperative tilt of IOLs is related to the preoperative tilt of the crystalline lens [16], the position of the crystalline lens before cataract surgery could differ in myopic eyes from that in nonmyopic eyes. This IOL tilt and decentration could negatively affect the postoperative optical performance. Therefore, a better understanding of the preoperative crystalline lens tilt in myopic and nonmyopic eyes could be useful for IOL selection and to predict the outcome of cataract surgery.

Two studies have assessed lens tilt using swept-source optical coherence tomography (SS-OCT), (IOLMaster 700, Carl Zeiss Meditec AG, Jena, Germany) [16, 17]. However, neither examined myopia as an important variable or systemically investigated how AL affects the magnitude or direction of crystalline lens tilt. Furthermore, these studies did not include many high myopic subjects or subjects with severe myopia.

In this study, we investigated the difference in lens tilt between subjects with age-related cataract and those with high-myopia-related cataract to provide more information on the IOL position. We used SS-OCT (IOLMaster 700) to measure the lens tilt and other intraocular parameters.

## Materials and methods

### Ethics statement

The Institutional Review Board of the Eye and ENT Hospital of Fudan University, Shanghai, China, approved this study (NO.2013021). All procedures adhered to the Declaration of Helsinki and were conducted in accordance with the approved protocol. Written informed consent was obtained from each patient before his/her participation.

### Patient selection

In this study, only one randomly chosen eye of each patient was analyzed. The exclusion criteria were previous eye surgery, including laser in situ keratomileusis [18], silicone oil tamponade [19], cataract surgery, and any procedure that may have interfered with intraocular structures, as well as eye conditions that impaired fixation, corneal opacities, or the presence of an IOL.

The subjects were then divided into three groups according to AL, as follows [9, 20]: emmetropia (AL < 24.5 mm; group E), mild/moderate myopia (AL 24.5–26 mm; group M), or high myopia (AL > 26 mm; group H). Group H was divided into three subgroups with AL of 26–28 mm (H1), 28–30 mm (H2), or > 30 mm (H3).

### Lens tilt measurement

SS-OCT was used to measure the eye parameters without pupil dilation during outpatient visits. The IOLMaster 700 is a noncontact optical biometer that measures eye structures and performs IOL power calculations with a deeper range of imaging, less sensitivity reduction with depth, and faster scanning speed than similar SS-OCT devices like Lenstar and IOLMaster 500 [21, 22]. The IOLMaster 700 measures the thickness and depth along the visual axis, providing full-eye tomograms at 2000 A-scans per second. It also provides biometric data with B-scan technology and configures the central 1.0 mm vitreoretinal interface of the examined eye [16].

### Data analysis

The raw data obtained with SS-OCT were analyzed with prototype purpose-designed software (Carl Zeiss Meditec AG) to determine the lens tilt. During the measurements, SS-OCT performed six B-scans at 0°, 30°, 60°, 90°, 120°, and 150°, and all the scans were repeated three times in this six-meridian pattern [17]. Parabolas were fitted to the cornea and lens surface on all 18 B-scans per eye and were dewarped to compensate for the geometric distortions caused by the scanning geometry and refraction in the eye [16]. The 18 two-dimensional (2D) mean parabolas were then transferred to a common 3D coordinate system, with compensation for lateral motion. Multiple linear regression was then performed to fit a plane to the 3D points sampled from the 18 transferred mean parabolas, and the orientation of the plane described the orientation of the tilt of the lens.

**Fig. 1:**
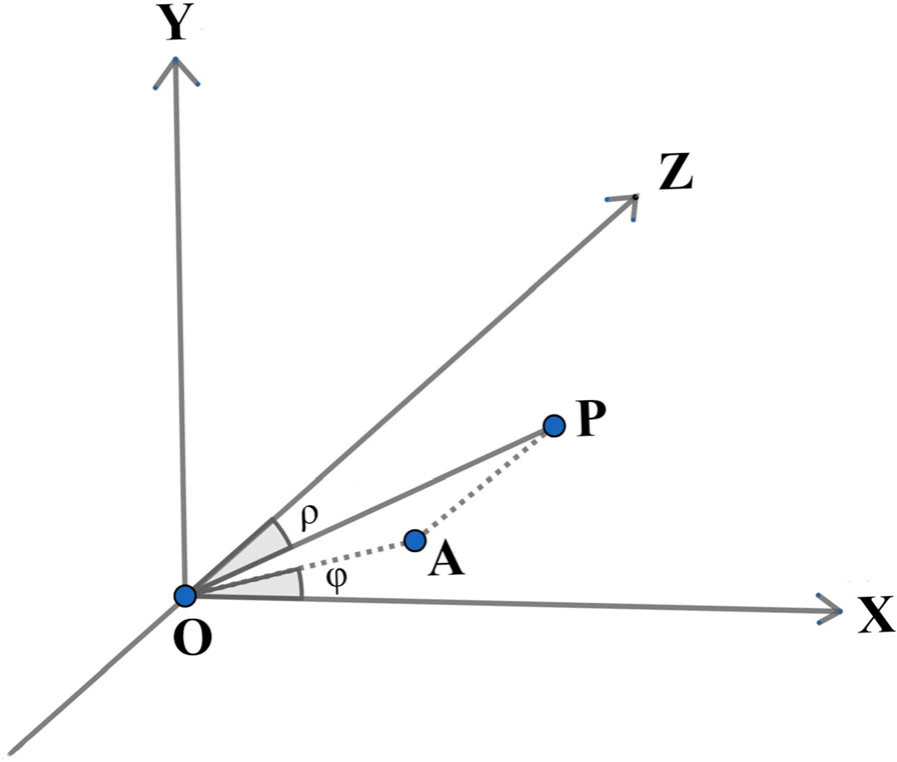
Schematic diagram of the calculation of lens tilt in the space rectangular coordinate system (O-XYZ). OP is the unit normal vector for the lens plane. OA is the projection of OP on the X–Y plane. Angle *ρ* is the angle between OP and the Z-axis and represents the magnitude of the lens tilt. Angle *φ* is the angle between OA and the X-axis, and represents the direction of the lens tilt. X-axis: horizontal axis; Y-axis: vertical axis; Z-axis: visual axis

On the 3D reconstruction of the lens in this space rectangular coordinate system (O-XYZ) (Fig. 1), the lens unit normal vector is represented as OP (*n*_x_, *n*_y_, *n*_z_), which is a line perpendicular to the lens surface joining the apices of the anterior and posterior lens surfaces. OP is an essential indicator of the magnitude and direction of the lens tilt. The tilt magnitude is the angle between OP and the visual axis (OZ), ranging from 0° to 90°, and is called angle *ρ*. OA is the projection of OP on the X–Y plane, and angle *φ* is the angle between OA and the X-axis. Angle *φ* represents the tilt direction in this study and ranges from − 180° to 180°. Angles *ρ* and *φ* are defined as follows:

$$ \rho =\mathrm{acos}\ \left({n}_{\mathrm{z}}\right) $$

$$ \varphi =\mathrm{atan}2\ \left({n}_{\mathrm{y}},{n}_{\mathrm{x}}\right) $$

Due to the nonsuperimposable mirror-image symmetry of human eyes (enantiomorphism), the lens tilt directions were categorized as outward/inward and upward/downward, thus dividing the tilt orientation into four quadrants. When OA was in the same direction as the positive direction of the Y-axis, *φ* was defined as − 90°. Therefore, if OA was in the opposite position, *φ* was 90°. When OA was in the same direction as the positive direction of X axis, *φ* was 0°, with values of 180° or − 180° in the opposite situation. Since vector OP is always oriented into the eye, the opposite to OP is where the anterior surface of the crystalline lens faces. The orientation of the lens tilt was then divided into four quadrants e.g., the ‘upper outer quadrant’ means that the lens surface faces outwards, towards the temporal side, and upwards. The other quadrants were deduced accordingly.

We also assessed other ocular parameters automatically provided by IOLMaster 700, including AL, anterior chamber depth (ACD, which refers to the distance from the corneal endothelium to the anterior lens capsule in this study), central corneal thickness (CCT), lens thickness (LT), and total keratometry of the corneal surface. These data were compared among the groups of subjects with different AL, and their correlations with crystalline lens tilt were determined.

### Statistical analysis

All statistical analyses were performed with SPSS version 13.0 (SPSS Inc., Chicago, IL, USA). Quantitative data are presented as means ± standard deviations, and the corresponding ranges are also provided. The Shapiro–Wilk test was used to test the normality of variables and the Levene test was used to examine the homogeneity of variance. A χ^2^ test was used to evaluate categorical variables. Student’s *t*-test was used to compare continuous parametric variables between two groups, and one-way analysis of variance (ANOVA) followed by Tukey’s post hoc test was used to compare three or more groups of patients. Pearson’s correlation and a linear regression analysis were used to determine the correlations between selected variables. *P* values of less than 0.05 were considered statistically significant.

## Results

### Patient characteristics

**Fig. 2:**
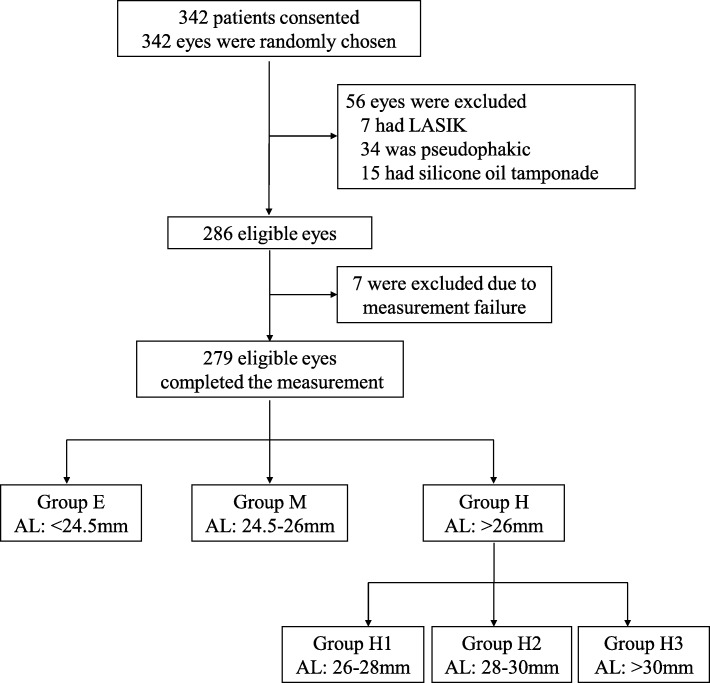
Patient flow

As shown in Fig. 2, 342 patients scheduled for cataract surgery at the outpatient clinic of the Eye and ENT Hospital of Fudan University between April 1 and July 30, 2018, with no other ophthalmological comorbidities, were initially enrolled. Of these, 63 patients were excluded. Therefore, 279 eyes of 279 patients were available for the final analysis. These subjects were divided into groups according to their AL. Table 1 lists the demographic data of the patients and the biometric data of the eyes enrolled in the study.

**Table 1:** Demographic and clinical data for patients with different axial lengths^a^

AL (mm)	< 24.5	24.5~26	26~28	28~30	> 30	*P* value^b^	Correlation with AL^c^
	21.40–24.49	24.51–25.90	26.02–27.97	28.12–29.92	30.08–37.96		
Subjects (n)	131	25	52	29	42	/	/
Right/left eye	68/63	14/11	21/31	15/14	25/17	0.425	/
ACD (mm)	2.34 ± 0.46 (1.16–3.39)	2.73 ± 0.31 (2.10–3.30)	2.93 ± 0.35 (2.07–4.38)			< 0.001^d^	r = 0.521 P < 0.001^d^
CCT (mm)	0.54 ± 0.03 (0.45–0.64)	0.56 ± 0.04 (0.51–0.70)	0.55 ± 0.04 (0.46–0.64)			0.027^d^	r = 0.094 P = 0.116
LT (mm)	4.51 ± 0.54 (3.25–5.81)	4.38 ± 0.49 (3.27–5.36)	4.34 ± 0.42 (3.28–5.79)			0.023^d^	r = − 0.116 *P* = 0.053
Flat K (D)	43.01 ± 1.54 (40.16–47.62)	42.02 ± 1.44 (39.09–44.61)	42.03 ± 1.50 (36.29–45.32)			< 0.001^d^	r = − 0.333 *P* < 0.001^d^
Steep K (D)	44.00 ± 1.61 (40.99–48.43)	43.02 ± 1.65 (39.33–45.56)	43.23 ± 1.71 (36.89–46.88)			< 0.001^d^	r = − 0.241 P < 0.001^d^
Average K (D)	43.51 ± 1.53 (40.61–48.04)	42.52 ± 1.52 (39.25–44.99)	42.63 ± 1.56 (36.59–45.54)			< 0.001^d^	r = − 0.293 P < 0.001^d^

*AL* = axial length; *ACD* = anterior chamber depth (from the corneal endothelium to the anterior lens capsule); *CCT* = central corneal thickness; *LT* = lens thickness; *K* = (conventional) keratometry

^a^ Data are presented as mean ± standard deviation; range is listed below in brackets

^b^ Differences among three groups [groups E (AL < 24.5 mm), M (AL 24.5–26 mm), and H (AL > 26 mm)] were analyzed with one-way ANOVA, and a χ^2^ test was used to compare categorical data

^c^ Correlations between selected variables and AL were analyzed with Pearson’s correlation test

^d^ Statistically significant (*P* < 0.05)

### Magnitude of lens tilt

We checked the mirror symmetry of the crystalline lens in our study and found no significant differences in the magnitude of lens tilt between the right and left eyes in each AL group (Student’s *t*-test, *P* > 0.1 in all cases). Therefore, we pooled the data for both eyes for the analysis.

**Fig. 3:**
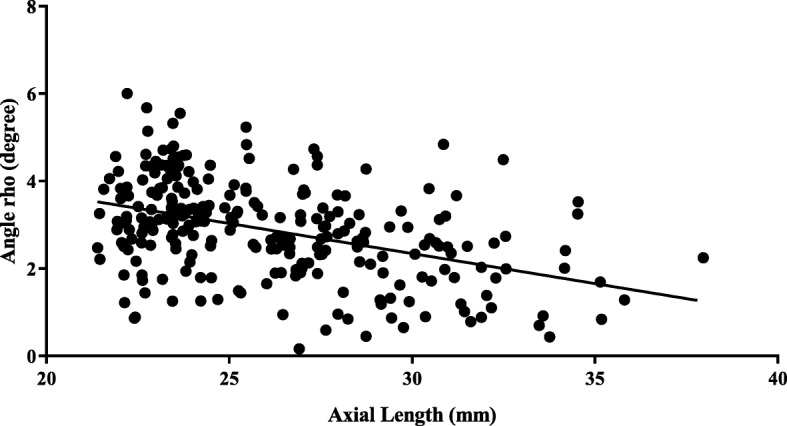
Correlation between axial length and angle *ρ*. The line of best fit was *y* = − 1.325*x* + 29.831 (*R* = 0.427, *P* < 0.001)

There were significant differences in the crystalline lens tilt among the five groups of subjects based on AL (one-way ANOVA, *P* < 0.001). Lens tilt differed significantly between group E and H1, H2, H3 as well as group H (Tukey’s post-hoc test, *P* < 0.001) (Table 2). Significant differences were also detected between groups H2, H3, and M (Table 2). The magnitude of tilt tended to decrease with increasing AL (Fig. 3). A linear regression analysis yielded the line of best fit *y* = − 1.325*x* + 29.831 (*R* = 0.427, *P* < 0.001). This inverse correlation was moderate, with Pearson’s *r* = − 0.427 (*P* < 0.001).

**Table 2:** Angle *ρ* in five groups with different axial lengths

Group	Lens tilt (°)		*P*-value^a^	*P*-value^b^
	Mean ± SD	Range		
E	3.36 ± 0.98	0.86–6.00	/	
M	3.07 ± 1.04	1.29–5.23	0.663	/
H	2.35 ± 1.01	0.17–4.84	< 0.001^c^	0.003^c^
H1	2.61 ± 0.97	0.17–4.73	< 0.001^c^	0.322
H2	2.19 ± 0.96	0.45° - 4.27	< 0.001^c^	0.012 ^c^
H3	2.15 ± 1.04	0.44° - 4.84	< 0.001^c^	0.003 ^c^

*E* = emmetropia; *M* = mild/moderate myopia; *H* = high myopia; *H1* = high myopia with axial length (AL) of 26–28 mm; *H2* = high myopia with AL of 28–30 mm; *H3* = high myopia with AL > 30 mm; *SD* = standard deviation

^a^ Differences among the three / five groups were analyzed with one-way ANOVA and Tukey’s post hoc test for multiple comparisons; the *P*-value here relates to the difference between the selected group and group E

^b^ Differences among the three / five groups were analyzed with one-way ANOVA and Tukey’s post hoc test. For multiple comparisons; *P*-value here showed difference between the selected group and group M

^c^ Statistically significant (*P* < 0.05)

### Correlations between other ocular biometric parameters and lens tilt

As shown in Table 1, ACD generally increased with increasing AL (Pearson’s *r* = 0.521, *P* < 0.001). CCT did not correlate significantly with AL (Pearson’s *r* = 0.094, *P* = 0.116). Although LT did not correlate significantly with AL (Pearson’s *r* = − 0.116, *P* = 0.053), significant differences in LT were observed among the three groups (one-way ANOVA, *P* = 0.023), with group H generally having thinner LT than group E (LT = 4.33 ± 0.42 mm and 4.51 ± 0.54 mm in groups H and E, respectively; Tukey’s post hoc test, *P* = 0.018).

With respect to the correlations between these parameters and lens tilt, Pearson’s correlation coefficients revealed that angle *ρ* correlated significantly with ACD (Pearson’s *r* = − 0.263, *P* < 0.001), but not with the other ocular parameters (Table 3).

**Table 3:** Correlations between the magnitude of lens tilt (angle *ρ*)^a^ and other ocular biometric parameters

	*r*	*P*
ACD	−0.263	< 0.001^b^
CCT	−0.004	0.948
LT	−0.040	0.504
Flat K	−0.044	0.473
Steep K	−0.070	0.254
Average K	−0.059	0.337

*ACD* = anterior chamber depth (from the corneal endothelium to the anterior lens capsule); *CCT* = central corneal thickness; *LT* = lens thickness; *K* = (conventional) keratometry

^a^ Correlations between selected variables and angle *ρ* were analyzed with Pearson’s correlation analysis, with Pearson’s r and *P* values presented

^b^ Statistically significant (*P* < 0.05)

### Orientation of lens tilt

**Fig. 4:**
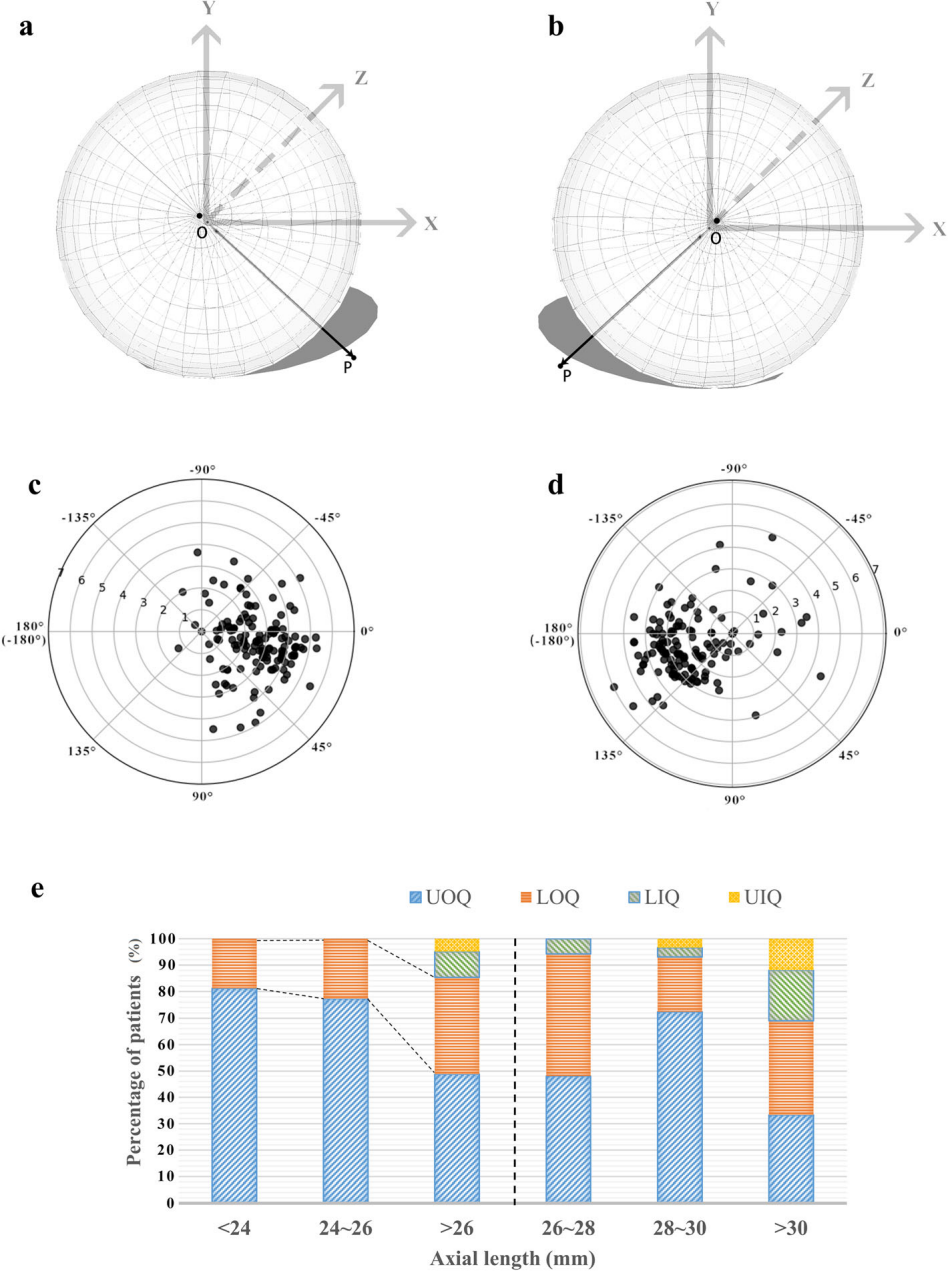
Orientation of crystalline lens tilt. (**a, b**) Schematic diagram of lens tilt in the right (a) and left eyes (b). Lenses from both eyes are facing towards the upper outer quadrants. A gray shadow was added for better visualization. (**c, d**) Polar scatterplots showing the magnitude (angle *ρ*) and orientation (angle *φ*) of lens tilt in the right (b) and left (c) eyes. Each ring represents 1°. (**e**) Stacked column graph of the lens tilt orientations in the different groups according to axial length. UOQ: upper outer quadrant; LOQ: lower outer quadrant; LIQ: lower inner quadrant; UIQ: upper inner quadrant

The tilt orientation was predominantly in the upper outer quadrant (Fig. 4a,b), which accounted for 94/143 of right eyes and 91/136 of left eyes, with mean tilt orientations (*φ*) of 24.32 ± 19.85° and 147.36 ± 23.77°, respectively (Fig. 4c,d). A tilt orientation in the lower outer quadrant was the second most frequent orientation, accounting for 44/143 and 32/136 of right and left eyes, respectively. The tilt orientation in the two inward quadrants was infrequent, and always found in eyes with long AL (χ^2^ test, *P* < 0.001). A tilt orientation in the lower inner quadrant was detected in three right eyes and nine left eyes, whereas a tilt in the lower upper quadrant was detected in two right eyes and three left eyes (Fig. 4e).

The tilt magnitude was greatest for lenses with a tilt direction in the upper outer quadrant (one-way ANOVA, *P* < 0.001), and decreased with the frequency of tilt orientation especially between the upper outer quadrant and the inner quadrants (Tukey’s post hoc test, *P* < 0.02).

## Discussion

Phacoemulsification and IOL implantation are routine procedures in cataract surgery. As surgical techniques and IOL design improve, a better understanding of ocular characteristics will improve IOL performance.

Zonular weakness and contraction of the capsular bag, which may exacerbate the weakness of the zonular fibers, are the two main reasons for in-the-bag IOL dislocation, and are more frequent in high myopic eyes after surgery [13, 23–25]. Zonular weakness is related to structural changes [25] and the proinflammatory status of the aqueous humor [13] in high myopic eyes, which may be present preoperatively. Because the preoperative crystalline lens position can help to predict the postoperative IOL status [16] and the tilt and decentration of an IOL can affect the surgical outcome, the preoperative evaluation of the crystalline lens position should be clinically useful.

In this study, we used the IOLMaster 700 to measure ocular parameters, including the position of the crystalline lens. Other methods have previously been used to measure IOL tilt and decentration, such as ultrasound biomicroscopy [26], anterior-segment OCT [27], and Purkinje and Scheimpflug imaging [28–30]. In those studies, measurements were made with reference to different planes or axes, including the iris plane, the corneal topographic axis, the pupillary axis, and the visual axis. The visual axis is defined as the line connecting the fixation point to the fovea, so the light path along this axis may directly reflect the visual perception [31]. In this study, whole-eye scanning with SS-OCT guaranteed a measurement along the visual axis. With a deeper imaging range, faster scanning speed, improved alignment of scans, and better illustration of the normal position of the crystalline lens with reference to the visual axis, the IOLMaster 700 ensures more satisfactory images and more-accurate measurements of the lens position.

To our knowledge, only two other studies have measured the lens tilt with the IOLMaster 700 [16, 17]. Their results could be useful as reference points for our study, in which we focused mainly on the characteristic of lens tilt in high myopic eyes.

In this study, the crystalline lens tilt was 3.36 ± 0.98° in group E and was smaller in the other groups with longer ALs. The magnitude of the lens tilt in group E was slightly smaller in this study than in previous studies (3.7 ± 1.1° [17] and 4.3 ± 0.9° [16]). Our finding that the magnitude of the tilt was smaller in high myopic eyes is consistent with a previous finding that corneal vertex inward displacement (or relative pupil outward displacement) is more likely in eyes with shorter AL and less likely in high myopic eyes [32].

The symmetrical direction of the crystalline lens tilt was demonstrated again in terms of magnitude in this study, and most lenses were tilted with an outward and upward orientation. This confirms the relative tilt of the optical axis when the eye is aligned along the visual axis [33]. This tilt orientation was predominant in eyes with a shorter AL, whereas greater variability in orientation was observed in high myopic eyes. This phenomenon could be explained by zonular weakness and inadequate structural support for the crystalline lens in high myopic eyes.

Intriguingly, although LT was not associated with myopia in a previous study [34], we detected a significant difference in LT between the high myopic group and the emmetropic group in this study and in another study using the IOLMaster 700 [17]. High myopic eyes are more susceptible to zonular failure [12, 35]; therefore, we suspect that the reduction in LT with longer AL can be attributed in part to zonular weakness. Zonular failure may cause the lens to sink, which will affect the accurate measurement of LT. This explanation may also apply to the magnitude and orientation of the lens tilt. In group M, the zonular fibers were fully stretched, holding the crystalline lens in an outward and upward direction, and thus maintaining the lens at a certain angle to the visual axis. However, in high myopic eyes the zonules may be too weak to hold the suspended crystalline lens in place, allowing it to be more affected by gravity and the head position, causing the lens to tilt in different directions and reducing the lens tilt.

Weak zonules could also result in the postoperative decentration and tilt of the IOL in cataract patients with high myopia, and reduce their postoperative visual quality [15, 36]. As well as applying capsular tension rings, the size, weight, and shape of the IOL could be improved to provide better surgical outcomes in high myopic eyes. Therefore, the idea of a further study on how the preoperative lens tilt is correlated with postoperatively IOL status and visual quality is appealing, and we plan to incorporate results from our study into clinical practice in the future.

## Conclusion

With the largest number of high myopic eyes included in this cohort of cataract patients examined with IOLMaster 700, the magnitude of the lens tilt showed significant negative correlation with increasing AL. In the majority of eyes, the crystalline lens faced the upper outer quadrant in both eyes, but the orientation showed greater variability with increasing AL, possibly indicating zonular weakness in high myopic eyes. We found a clear difference in the preoperative crystalline lens tilt between high myopic eyes and eyes with less myopia. This could be an important determinant of postoperative IOL performance. Therefore, preoperative lens analysis could be an essential stage in IOL selection, especially in high myopic eyes, which are at greater risk of lens tilt and decentration. For the sake of better surgical outcomes in high myopia group, IOLs with different size, weight and shape could be specially designed in the future.

## Data Availability

The datasets generated and/or analyzed during the present study are not publicly available (obtained from Eye and Ear, Nose, and Throat Hospital, Fudan University, Shanghai repository), but are available from the corresponding author upon reasonable request.

## Abbreviations

3D: Three dimensional
ACD: Anterior chamber depth
AL: Axial length
ANOVA: Analysis of variance
CCT: Central corneal thickness
IOL: Intraocular lens
LT: Lens thickness
